# Does Maternal Quality of Life Influence Breastfeeding Difficulties?

**Published:** 2014-07-20

**Authors:** Forough Mortazavi, Seyed-Abbas Mousavi, Reza Chaman

**Affiliations:** 1 Reproductive Health, Sabzevar University of Medical Sciences, Sabzevar; 2 Department of psychiatry, Research Center of Psychiatry, Golestan University of Medical Sciences, Golestan; 3 Department of Epidemiology, School of Medicine, Yasuj University of Medical Sciences, Yasuj, Iran

**Keywords:** Breastfeeding Difficulties, Quality of Life, Postnatal, Pregnancy

There is extensive evidence for short-term and long-term health benefits of breastfeeding for mothers and babies^[^^1^^]^. However, for some women coping with common breastfeeding problems in early postpartum is a physically and emotionally exhausting task, and they may harbor doubts about the continuation of breastfeeding^[^^2^^]^. A study conducted in Iran has reported that difficulties such as sore nipples or the mother's perception of having insufficient milk were the reasons for the exclusive breastfeeding discontinuation^[^^3^^]^. The mother’s perception and reaction to breastfeeding difficulties are affected by multiple factors, such as psychological and physical health, socio-demographic characteristics, quality of marital relationship and living condition^[^^4^^]^. Quality of life (QOL) is a broad ranging concept that includes all the mentioned aspects^[^^5^^]^. Therefore, the present study aims to investigate the relationships between QOL and breastfeeding difficulties in a sample of mothers from northeast Iran. 

 358 women who attended urban health centers, agreed to participate in the study. The inclusion criterion was gestational age of at least 28 weeks. The participants completed the World Health Organization Quality of Life - brief version (WHOQOL-BREF) in the third trimester of pregnancy. It contains 24 questions divided between 4 domains: Physical, Psychological, Social Relationships and Environment^[^^5^^]^. The validity and reliability of the Iranian version of WHOQOL-BREF have been supported in a previous study^[^^6^^]^. Women completed the Breastfeeding Experience Scale (BES) at 4 weeks postpartum. The first 18 questions of the BES rate the severity of common breastfeeding difficulties using a 5‐point Likert scale. Content validity and internal consistency of this scale (alpha coefficient 0.76) has been supported in a previous study^[^^7^^]^. In our study, the alpha coefficient was 0.82. 

 Mean age of women was 26.17 and 58.7% of them were primigravida. The mean total score of the breastfeeding difficulties questionnaire was 31.4±8.5. Common difficulties experienced by women were baby nursing too frequently (81.6%), difficulty in combining housekeeping and breastfeeding (69%), feeling very tired or fatigued (59%), and worry about having enough milk (52%). The correlation coefficients between breastfeeding difficulties score and the score of physical, mental, social, environmental, and global score of QOL were -0.217 (*P*<0.001), -0.172 (*P*=0.001), -0.157 (*P*=0.004), -0.154 (*P*=0.004), and -0.168 (*P*=0.002) respectively. Multiple regression analysis controlling for the effects of confounder variables showed that the global score of QOL was a predictor of breastfeeding difficulties (B= -0.21, CI [-0.20, -0.07]). The variables remained in the model explained 22.3% of the variance in breastfeeding difficulties (F=10.1, *P*=0.002). We explored differences of breastfeeding difficulties means according to different levels of satisfaction revealed in the independent Q1 of the WHOQOL-BREF, which asks about an individual’s overall perception of QOL. The mean breastfeeding difficulties score (SD) according to each level of satisfaction was as follows: ‘poor’ or ‘neither poor nor good’, 32.8 (8.5); ‘good’, 31.8 (8.5); and ‘’very good, 29.6 (8.5). There was a significant difference of breastfeeding difficulties scores according to 3 levels of perception of quality of life (F=3.15, *P*=0.04).

 Our results indicate that there is a weak and negative correlation between quality of life scores and breastfeeding difficulties scores. However, prenatal QOL was related independently to breastfeeding difficulties. Mothers with poor QOL are more likely to experience breastfeeding difficulties in early postpartum. These findings are comparable with a Brazilian research, which found a correlation between breastfeeding self-efficacy and maternal QOL^[^^8^^]^. In addition, we found that women who perceived their QOL as ‘good’ or ‘very good’ had lower breastfeeding difficulties scores than mothers who perceived their QOL as ‘not bad, not good’. 

 QOL was independently related to breastfeeding difficulties. Mothers with poor QOL are more likely to experience breastfeeding difficulties in early postpartum. To promote EBF, mothers with low QOL should be supported during the early postpartum. 
